# Pediatric ependymoma in the molecular era: real-world experience with diagnostic correlation and long-term clinical outcomes

**DOI:** 10.1007/s11060-026-05740-y

**Published:** 2026-08-05

**Authors:** Katerina Trkova, Ales Vicha, David Sumerauer, David R. Ghasemi, Vijay Ramaswamy, Lenka Krskova, Miroslav Koblizek, Josef Zamecnik, Martin Kyncl, Barbora Ondrova, Lucie Stolova, Marie Rybkova, Katarina Horcicakova, Vladimir Benes, Stefan Rutkowski, Michal Zapotocky

**Affiliations:** 1 Prague Brain Tumor Research Group, Second Faculty of Medicine, Charles University Prague and Motol and Homolka University Hospital, V Uvalu 84, Prague, Czech Republic; 2 Center for Pediatric Neuro-Oncology, Motol and Homolka University Hospital, Prague, Czech Republic; 3 Department of Pediatric Hematology and Oncology, Second Faculty of Medicine, Charles University Prague and Motol and Homolka University Hospital, Prague, Czech Republic; 4 Department of Pediatric Hematology and Oncology, University Medical Center Hamburg-Eppendorf, Hamburg, Germany; 5 Research Institute Children’s Cancer Center, Hamburg, Germany; 6 Mildred Scheel Cancer Career Center HaTriCS4, University Medical Center Hamburg-Eppendorf, Hamburg, Germany; 7 Hopp Children’s Cancer Center Heidelberg (KiTZ), Heidelberg, Germany; 8 Development and Stem Cell Biology, Labatt Brain Tumour Research Centre, Sickkids Research Institute, Toronto, ON Canada; 9 Department of Medical Biophysics and Paediatrics, University of Toronto, Toronto, ON Canada; 10 Division of Haematology/Oncology, Hospital for Sick Children, Toronto, ON Canada; 11 Department of Pathology and Molecular Medicine, Second Faculty of Medicine, Charles University Prague and Motol and Homolka University Hospital, Prague, Czech Republic; 12 Department of Radiology, Second Faculty of Medicine, Charles University Prague and Motol and Homolka University Hospital, Prague, Czech Republic; 13 Proton Therapy Center Czech, Prague, Czech Republic; 14 Department of Neurosurgery, Second Faculty of Medicine, Charles University Prague and Motol and Homolka University Hospital, Prague, Czech Republic

**Keywords:** Ependymoma, DNA methylation profiling, Molecular classification, Reclassification, Gross-total resection, Children

## Abstract

**Purpose:**

Real-world longitudinal data documenting the diagnostic impact of DNA methylation array (MA) profiling in pediatric ependymoma remain sparse. We report a single-center retrospective analysis evaluating MA-driven reclassification, molecular subgroup distribution, and long-term survival outcomes in a national pediatric referral cohort.

**Methods:**

Sixty-three pediatric patients with a histological ependymoma diagnosis treated at Motol and Homolka University Hospital (2010–2025) were included. DNA methylation profiling, RNA sequencing, copy number variation and t-SNE analysis were performed. Survival was estimated by the Kaplan–Meier method.

**Results:**

MA confirmed ependymoma in 48 patients (76.2%) and reclassified 15 (23.8%) as non-ependymoma entities, including newly described tumor types. The reclassification rate was 36.1% in the pre-2019 cohort versus 7.4% post-2019. Posterior fossa group A (PFA) ependymoma was the predominant subgroup (*n* = 26). Ten-year overall survival (OS) was 69.6% and event-free survival (EFS) 47.7%. Gross total resection was the only factor significantly associated with improved survival (OS 75% vs. 40%, *p* = 0.017). Chromosome 1q gain, identified exclusively in PFA patients, was associated with a high relapse rate despite standard therapy.

**Conclusion:**

MA-driven reclassification affected nearly one quarter of patients, with substantially higher rates in the pre-molecular era, confirming that integrated molecular diagnosis is indispensable in all pediatric CNS tumors referred with a histological diagnosis of ependymoma.

**Supplementary Information:**

The online version contains supplementary material available at https://doi.org/10.1007/s11060-026-05740-y.

## Introduction

Ependymomas represent a clinically and biologically heterogeneous group of glial tumors arising throughout the entire neuraxis, constituting the third most common malignant brain tumor and the most common spinal cord tumor in children [1–3]. For decades, their classification relied exclusively on histopathological features, a framework limited in prognostic value and limited by poor reproducibility problems: landmark analyses demonstrated that grading resulted in substantial inter-observer variability and lacked consistent clinical utility, particularly in the pediatric posterior fossa setting [4–6]. The introduction of DNA methylation profiling transformed this landscape profoundly, culminating in the 5th edition of the WHO Classification of CNS Tumors (2021), which recognized molecularly defined subgroups distributed across the supratentorial, posterior fossa, and spinal compartments, each with distinct biological drivers, age preferences, and prognostic implications [5, 7, 8]. This shift represented arguably the most significant reclassification of ependymoma since the first WHO classification in 1979 [1].

The clinical consequences of molecular subgroup assignment extend well beyond nomenclature. Subgroups differ substantially in prognosis — with posterior fossa group A (PFA) and supratentorial ZFTA fusion-positive (ST-ZFTA) ependymoma carrying the worst intracranial outcomes, while posterior fossa group B (PFB) and supratentorial YAP1 fusion-positive (ST-YAP1) ependymoma confer considerably more favorable trajectories [5, 9]. It is now well established that integrated diagnosis incorporating DNA methylation profiling is essential for accurate tumor classification in this setting, and its routine implementation has been endorsed as the diagnostic gold standard by the 2021 WHO Classification [7]. At the same time, thanks to new molecular methods, new biological subgroups of tumors have been described that may resemble ependymomas histologically but constitute distinct entities. These include, for example Neuroepithelial tumor, PLAGL1-fused; Neuroepithelial Tumor with PATZ1 Fusion and CNS BCOR-Altered Tumors [10–14].

Despite these advances, real-world longitudinal data from individual national reference centers documenting the diagnostic evolution across the transition from histology-only to integrated molecular diagnosis remain sparse. Such institutional datasets are of particular value because they capture the full spectrum of diagnostic uncertainty encountered in routine clinical practice, and allow direct comparison of diagnostic accuracy across the pre- and post-methylation-profiling eras — a perspective that large consortium-level studies are less suited to provide.

Here, we report a retrospective single-center analysis of 67 pediatric patients with a histological diagnosis of ependymoma managed at a national referral center between 2010 and 2025. Spanning both the pre- and post-methylation-profiling diagnostic eras, we describe the molecular subgroup distribution across anatomical compartments, the frequency and nature of MA-driven reclassification, its temporal evolution, and long-term survival outcomes stratified by molecular subgroup and extent of surgical resection.

## Methods

### Patient cohort and tumor samples

This is an observational study. The Institutional Ethics Committee of the Motol and Homolka University Hospital, Prague has confirmed that no ethical approval is required. Written informed consent for the use and publication of anonymized clinical and molecular data was obtained from the legal representatives of all participating patients. All analyses were performed in compliance with applicable ethical guidelines and institutional regulations.

Patients with a histological diagnosis of ependymoma treated at our center between 2010 and 2025 were identified through institutional records. Medical charts were reviewed to obtain demographic data, tumor location, primary histological diagnosis, and clinical course at the time of diagnosis. Archival formalin-fixed paraffin-embedded (FFPE) tissue blocks and/or frozen tissue samples were retrieved for all available cases and used for subsequent molecular analyses as detailed below.

### Treatment

The primary therapeutic goal for all patients was achieving gross total resection (GTR). In patients older than 12 months, surgery was followed by focal photon or proton radiotherapy 54–59,6 Gy with 5 mm clinical target volume margin and 3–5 mm planning target volume margin according to institutional protocols. When GTR could not be achieved at initial surgery, several cycles of neoadjuvant ACNS0121 or SIOP Ependymoma II protocol based chemotherapy were administered with the aim of achieving GTR at second-look surgery [15, 16]. In very young children (< 12 months), treatment consisted of chemotherapy alone, using either the chemotherapy regime based on UKCCSG CNS 9204 protocol or SIOP Ependymoma II protocol [16, 17].

Management of relapsed disease was individualized, with the goal of achieving local control through resection combined with radiotherapy and chemotherapy.

### RNA and DNA extraction

Nucleic acids were extracted from formalin-fixed, paraffin-embedded (FFPE) tissue blocks using the QIAamp DNA FFPE Tissue Kit (Qiagen, Germany) for genomic DNA and the High Pure RNA Paraffin Kit (Roche Diagnostics, Mannheim, Germany) for total RNA. In cases where fresh-frozen tissue was available, both genomic DNA and total RNA were extracted using TRIzol Reagent (Life Technologies, Merelbeke, Belgium). Prior to nucleic acid isolation, a neuropathologist selected the most representative tissue block containing the maximum proportion of viable tumor tissue.

### RNA NGS and RT-PCR

RNA sequencing libraries were prepared from FFPE or fresh-frozen tumor RNA using the FusionPlex Oncology Research Kit (ArcherDX) and/or the FusionPlex PanSolid Tumor V2 Kit, and sequenced on a MiSeq instrument (Illumina). Data were analyzed using Archer Analysis and Arriba software (https://github.com/suhrig/arriba/). Fusion transcripts identified by RNA sequencing were validated by RT-PCR, with RNA reverse-transcribed using MMLV reverse transcriptase (Gibco BRL, Carlsbad, TX, USA) and PCR performed using PCRBIO HS Taq Mix Red (PCR Biosystems Ltd., London, UK).

### Methylation array, copy number profiling and t-SNE analysis

Genome-wide DNA methylation profiling was performed using the Infinium MethylationEPIC (EPIC) BeadChip (Illumina, San Diego, CA, USA) according to the manufacturer’s instructions and as previously described [18]. Both FFPE and fresh-frozen tissue samples were used as input material. All computational analyses were performed in R version 3.6.0 (R Development Core Team, 2016; https://www.R-project.org). Copy number variation (CNV) analysis was performed using the conumee Bioconductor Package (version 1.12.0), and images were captured on a NextSeq 550 instrument (Illumina, San Diego, CA, USA). Tumor classification and CNV analysis were performed using the CNS tumor classifier version 12.8 (https://www.https://epignostix.com/).

The clustering visualization via t-Distributed Stochastic Neighbor Embedding analysis (t-SNE) was performed as previously described [5, 18, 19]. Briefly, unsupervised non-linear dimension reduction was performed by calculating the 1-variance weighted Pearson correlation between samples after filtering for probes with single-nucleotide polymorphisms spanning five base pairs of and including the targeted CpG-sites, probes not mapping uniquely to the human reference genome, and probes that mapped to the X and Y chromosomes. The resulting distance matrix was used as input for t-SNE using the Rtsne package version 0.13. The following non-default parameters were applied: theta = 0, pca = F, max_iter = 2500, perplexity = 20. The reference cohorts were taken from previously published studies [5, 8].

### Statistical analysis

Overall survival was estimated using the Kaplan–Meier method, with between-group differences evaluated by the log-rank test. All statistical calculations were carried out in R (version 4.1.2; R Development Core Team).

## Results

### Patient cohort

Between 2010 and 2025, 67 patients with a histological diagnosis of ependymoma were enrolled at our center (Table 1). This group of patients constitutes a consecutive cohort that covers more than two-thirds of the population of the Czech Republic.

Methylation array (MA) analysis was performed in 63 patients with available material. The 4 patients for whom material was not available did not differ from the cohort characteristics (2 patients with spinal ependymoma, one with supratentorial and one with infratentorial localization). Further analysis will only concern patients with available methylation array results. The cohort consisted of 33 female and 30 male patients. The median age at diagnosis was 6.2 (0.1–19.8). The cohort comprised 36 patients diagnosed before 2019 and 27 diagnosed from 2019 onwards, reflecting the two distinct diagnostic eras defined by the routine availability of MA at our center.

**Table 1:** Demographic, histopathology and molecular-biology data of the patients

year of DX	age at Dx	gender	Study_ID	histology	localization	methylation class	score	molecular biology
2010	19	f	NE13	ependymoma grade 3	supratentorial	Diffuse Glioma, IDH Mutant	0.99	IDH1 R132H
2010	11	f	NE14	ependymoma grade 3	posterior fossa	Diffuse Midline Glioma, H3k27-Altered	0.94	
2010	8	m	NE2	ependymoma grade 3	posterior fossa	Medulloblastoma Non-WNT/Non-SHH Activated	0.99	
2010	9	f	NE3	ependymoma grade 3	supratentorial	Diffuse Pediatric-Type High-Grade Glioma, H3-Wildtype And IDH-Wildtype	0.37	
2010	10	f	PRG45	ependymoma grade 3	posterior fossa	Posterior Fossa Ependymoma Group B	0.28	
2010	3	m	PRG9	ependymoma grade 2	posterior fossa	Posterior Fossa Ependymoma Group A	0.99	gain 1q
2011	1	f	NE10	ependymoma grade 3	supratentorial	Neuroepithelial tumour, PLAGL1-fused	0.93	EWSR1::PLAGL1
2011	15	f	NM4	ependymoma grade 3	posterior fossa	N/A		
2011	3	f	PRG10	ependymoma grade 3	posterior fossa	Posterior Fossa Ependymoma Group A	1	
2012	6	m	NE5	ependymoma grade 3	posterior fossa	Medulloblastoma Non-WNT/Non-SHH Activated	0.93	
2012	0	f	NM3	tanycytic ependymoma grade 2	spine	N/A		
2012	2	f	PRG16	ependymoma grade 2	posterior fossa	Posterior Fossa Ependymoma Group A	0.99	
2012	1	m	PRG18	ependymoma grade 3	supratentorial	Supratentorial Ependymoma, ZFTA Fusion-Positive	0.99	ZFTA::MAML2
2013	6	m	NE7	ependymoma grade 2	supratentorial	Neuroepithelial Tumour With PATZ1 Fusion	0.99	
2013	1	f	PRG20	ependymoma grade 3	posterior fossa	Posterior Fossa Ependymoma Group A	0.98	
2013	14	f	PRG44	ependymoma grade 3	posterior fossa	Posterior Fossa Ependymoma Group A	0.92	gain1q
2013	7	f	PRG8	ependymoma grade 3	supratentorial	Supratentorial Ependymoma, ZFTA Fusion-Positive	0.99	ZFTA::RELA
2014	7	m	NE6	ependymoma grade 3	posterior fossa	Medulloblastoma Non-WNT/Non-SHH Activated	0.99	iso17q
2014	5	f	NE9	ependymoma grade 3	posterior fossa	Medulloblastoma Non-WNT/Non-SHH Activated	0.95	
2014	5	m	NM2	ependymoma grade 2	spine	N/A		
2014	13	f	PRG1	ependymoma grade 3	posterior fossa	Posterior Fossa Ependymoma Group A	0.99	gain 1q
2014	2	f	PRG22	ependymoma grade 3	supratentorial	Supratentorial Ependymoma, ZFTA Fusion-Positive	0.99	ZFTA::BAD::RELA,chromothripsis 11
2014	1	m	PRG23	ependymoma grade 3	posterior fossa	Posterior Fossa Ependymoma Group A	0.99	
2014	1	f	PRG24	ependymoma grade 3	posterior fossa	Posterior Fossa Ependymoma Group A	0.99	
2014	8	f	PRG7	ependymoma grade 3	supratentorial	Supratentorial Ependymoma, ZFTA Fusion-Positive	0.99	ZFTA::RELA
2015	15	m	NE1	ependymoma grade 2	posterior fossa	Low-Grade Glioneuronal Tumour	0.99	
2015	12	m	NE11	papilar ependymoma grade 2	spine	Pleomorphic Xanthoastrocytoma(-Like)	0.55	KANK1::NTRK2
2015	12	m	PRG2	MPE grade 2	spine	Myxopapillary Ependymoma	0.99	
2015	2	m	PRG25	ependymoma grade 2	posterior fossa	Posterior Fossa Ependymoma Group A	1	
2015	1	f	PRG28	ependymoma grade 3	supratentorial	Supratentorial Ependymoma, YAP1 Fusion-Positive	0.99	YAP1::MAMLD1
2015	9	m	PRG4	ependymoma grade 3	supratentorial	Supratentorial ependymoma, ZFTA Fusion-Positive	0.99	ZFTA::RELA, chromothripsis 11q
2016	11	f	NM1	ependymoma grade 2	supratentorial	N/A		
2017	9	f	NE8	ependymoma grade 3	posterior fossa	CNS BCOR-Altered Tumours	0.99	
2017	6	m	PRG19	ependymoma grade 3	posterior fossa	Posterior Fossa Ependymoma Group A	0.99	
2017	15	f	PRG3	MPE grade 1	spine	Spinal Ependymoma (myxopapillary-enriched)	0.99	
2017	1	m	PRG31	ependymal tumor - not specified	supratentorial	Supratentorial ependymoma, ZFTA Fusion-Positive	0.99	ZFTA::RELA
2017	10	f	PRG6	ependymoma grade 3	posterior fossa	Posterior Fossa Ependymoma Group A	0.99	
2018	14	f	NE4	ependymoma grade 2	supratentorial	CNS BCOR-Altered Tumours	0.95	EP300::BCOR
2018	8	m	PRG15	ependymoma grade 3	posterior fossa	Posterior Fossa Ependymoma Group B	0.67	
2018	6	m	PRG21	ependymoma grade 3	supratentorial	Supratentorial ependymoma, ZFTA Fusion-Positive	1	ZFTA::RELA
2019	1	f	NE12	ependymoma grade 2	posterior fossa	Pilocytic astrocytoma	0.99	KIAA1549::BRAF
2019	18	f	NE15	ependymoma grade 2	supratentorial	Neuroepithelial tumour, PLAGL1-fused	0.99	EWSR1::PLAGL1
2019	11	m	PRG12	ependymoma grade 3	posterior fossa	Posterior Fossa Ependymoma Group A	0.99	gain 1q at diagnosis, loss 6q in relapse
2019	5	m	PRG26	ependymoma grade 3	supratentorial	Supratentorial ependymoma, ZFTA Fusion-Positive	0.99	ZFTA::RELA, CDKN2A/B del
2020	5	f	PRG27	ependymoma grade 2	posterior fossa	Posterior Fossa Ependymoma Group A	0.97	
2020	1	m	PRG32	ependymoma grade 3	posterior fossa	Posterior Fossa Ependymoma Group A	0.99	
2020	2	f	PRG34	ependymoma grade 3	posterior fossa	Posterior Fossa Ependymoma Group A	0.99	
2020	1	m	PRG35	ependymoma grade 3	supratentorial	Supratentorial Ependymoma, ZFTA Fusion-Positive	0.99	ZFTA::RELA, CDKN2A/B del, chromothripsis 11
2020	0	m	PRG36	ependymoma grade 3	posterior fossa	Posterior Fossa Ependymoma Group A	0.99	
2020	0	f	PRG37	ependymoma grade 2	supratentorial	Supratentorial Ependymoma, YAP1 Fusion-Positive	0.99	YAP1::MAMLD1
2020	0	m	PRG47	ependymoma grade 3	posterior fossa	Posterior Fossa Ependymoma Group A	0.99	
2021	11	m	PRG17	intramedular ependymoma grade 2	spine	Ependymal Tumours	0.85	
2021	1	m	PRG38	ependymoma grade 3	posterior fossa	Posterior Fossa Ependymoma Group A	0.99	
2021	0	m	PRG39	ependymoma grade 3	posterior fossa	Posterior Fossa Ependymoma Group A	0.99	
2022	14	f	PRG14	ependymoma grade 2	posterior fossa	Posterior Fossa Ependymoma Group B	0.66	
2022	6	f	PRG30	ependymoma grade 3	posterior fossa	Posterior Fossa Ependymoma Group A	0.98	gain 1q
2022	5	f	PRG46	ependymoma grade 2	supratentorial	Supratentorial ependymoma, ZFTA Fusion-Positive	0.99	ZFTA::RELA
2023	7	f	PRG29	ependymoma grade 2	posterior fossa	Posterior Fossa Ependymoma Group B	0.99	
2023	2	m	PRG40	ependymoma grade 3	posterior fossa	Posterior Fossa Ependymoma Group A	0.99	
2023	2	f	PRG42	ependymoma grade 3	posterior fossa	Posterior Fossa Ependymoma Group A	0.99	
2023	0	f	PRG43	ependymoma grade 3	supratentorial	Supratentorial Ependymoma, YAP1 Fusion-Positive	0.99	YAP1::MAMLD1
2023	2	f	PRG48	ependymoma grade 2	posterior fossa	Posterior Fossa Ependymoma Group A	0.99	
2023	16	m	PRG5	spinal ependymoma	spine	Spinal Ependymoma	0.99	
2024	15	m	PRG11	MPE grade 2	spine	Myxopapillary Ependymoma	0.99	
2024	16	m	PRG13	MPE grade 2	spine	Spinal Ependymoma (myxopapillary-enriched)	0.99	
2024	5	m	PRG33	ependymoma grade 3	posterior fossa	Posterior Fossa Ependymoma Group A	0.99	
2024	0	f	PRG41	ependymoma grade 3	posterior fossa	Posterior Fossa Ependymoma Group A	0.99	

### Molecular classification

MA confirmed the diagnosis of ependymoma in 48 patients (76.2%) and reclassified 15 patients (23.8%) as harboring a non-ependymoma entity (Table 1). Calibrated methylation score in the 12.8 classifier version in vast majority of ependymomas was > 0,9 (*n* = 44), equal 0,85 (*n* = 1), < 0,85 (*n* = 3); in non-ependymoma group was methylation calibrated score > 0,99 (*n* = 13), < 0,85 (*n* = 2).

By localization, confirmed ependymomas arose in the posterior fossa (*n* = 29), supratentorially (*n* = 13), and in the spine (*n* = 6). Among confirmed ependymomas, molecular subgroup assignment identified posterior fossa group A (PFA, *n* = 26), supratentorial ZFTA fusion-positive (ST-ZFTA, *n* = 10), posterior fossa group B (PFB, *n* = 3), supratentorial YAP1 fusion-positive (ST-YAP1, *n* = 3), myxopapillary ependymoma (MPE, *n* = 3), and spinal ependymoma (*n* = 2), with one case remaining subgroup-unassigned despite confirmed ependymoma classification.

A canonical fusion *ZFTA::RELA* was detected in eight patient with ST-ZFTA ependymoma, less common variants *ZFTA::MAML2* fusion and *ZFTA::BAD::RELA* were detected in two patients, respectively, from this group. In patients with ST-YAP1 ependymomas, we detected a fusion *YAP::MAMLD1* in three patients.

### CNV changes

CNV analysis focused on alterations currently associated with adverse prognosis in the literature: gain of chromosome 1q, loss of 6q, and *CDKN2A/B* deletion [20–22]. Gain 1q was detected in 5 patients, all with posterior fossa ependymoma group A (PFA) subtype. Four of five patients (80%) with gain 1q experienced relapse. Three of these four relapsed patients had received optimal primary treatment with GTR and radiotherapy, while one patient achieved only near-total resection, representing an additional negative prognostic factor. *CDKN2A/B* deletion was identified in 2 patients with ZFTA fusion-positive supratentorial ependymomas. One patient with *CDKN2A/B* deletion relapsed after primary treatment with GTR and chemotherapy according to the SIOP Ependymoma II protocol.

### Reclassified cases

The reclassified tumors were located in the posterior fossa (*n* = 8), supratentorial region (*n* = 6), and spine (*n* = 1) (Table 1). The first category comprises 10 patients in whom MA identified tumor entities that were already recognized nosological categories at the time of original diagnosis, but whose morphological features overlapped sufficiently with ependymoma to challenge histology-based classification: medulloblastoma non-WNT/non-SHH (*n* = 4), diffuse midline glioma H3K27-altered (*n* = 1), diffuse pediatric-type high-grade glioma H3-wildtype/IDH-wildtype (*n* = 1), IDH-mutant diffuse glioma (*n* = 1), pilocytic astrocytoma (*n* = 1), pleomorphic xanthoastrocytoma (*n* = 1), and low-grade glioneuronal tumor (*n* = 1). The second category comprises 5 patients reclassified as tumor types that had not yet been defined as distinct diagnostic entities at the time of diagnosis: CNS BCOR-altered tumor (*n* = 2), PLAGL1-fused neuroepithelial tumor (*n* = 2), and PATZ1-fused neuroepithelial tumor (*n* = 1). In these cases, these are new biological entities where the original histological designation reflected the limits of contemporary classification rather than a diagnostic oversight.

### t-SNE analysis results

**Fig. 1:**
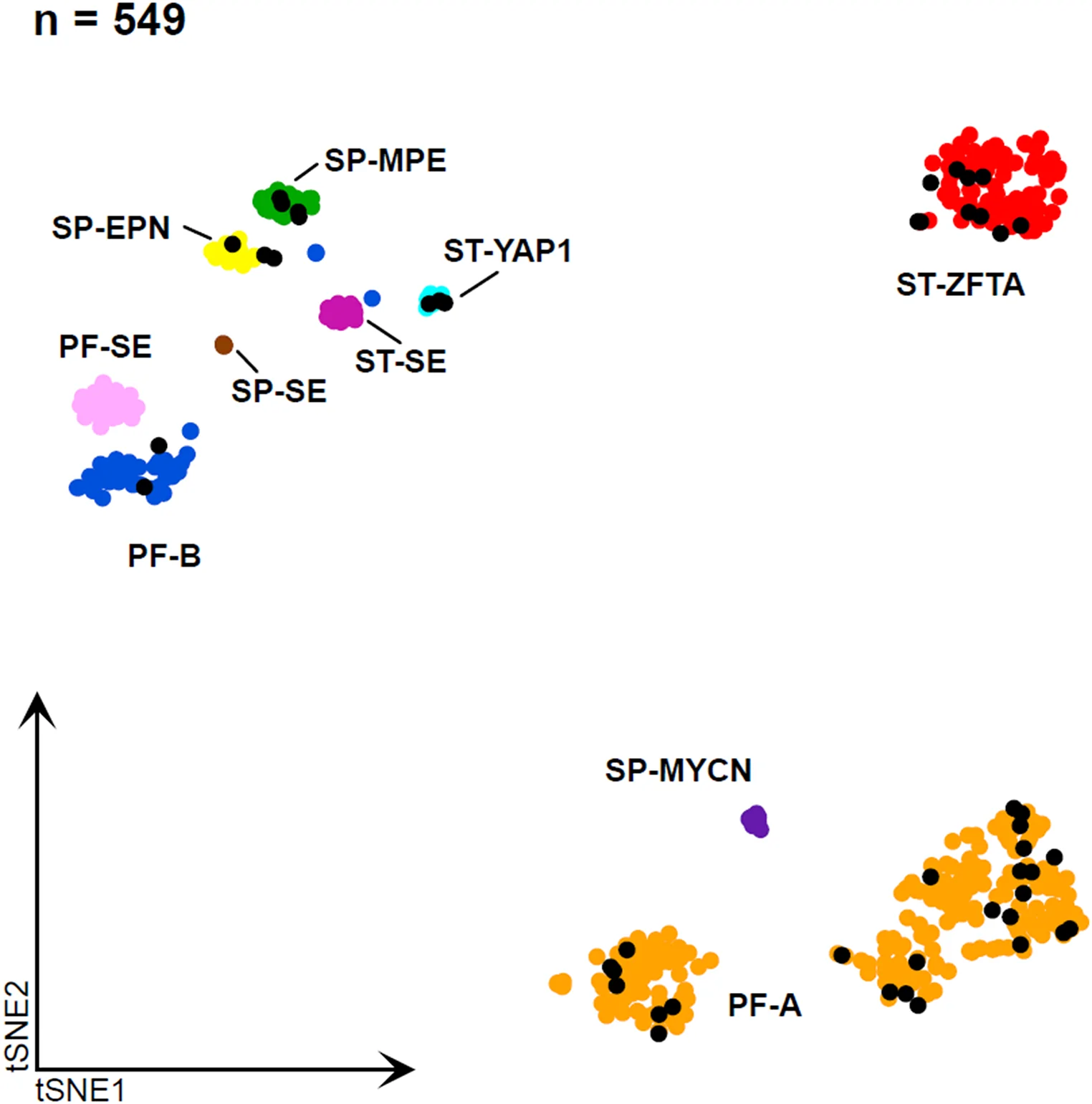
t-SNE analysis of current cohort patients compared to previously published studies

t-SNE analysis was performed using a reference cohort and demonstrated that all our samples clustered with previously described ependymoma subgroups (Fig. 1). However, the t-SNE analysis helped biologically classify one sample with low PFB score with aggressive course of the disease which clusters with PFA group. 

### Impact of MA on diagnostic accuracy over time

The reclassification rate differed substantially between the two study periods. Among patients diagnosed before 2019, MA reclassified 13 of 36 patients (36.1%) as non-ependymoma.

It is of note that in 9 histologically misclassified cases, the diagnosis had already been questioned by the clinical team, based on features such as metastatic relapse more typical of medulloblastoma, unexpectedly rapid progression, or atypical MRI findings — clinical suspicion that arose only later in the disease course rather than at diagnosis. MA those retrospectively confirmed and molecularly characterized what had been clinically suspected. However, other misclassifications were identified by MA in patients with clinically unremarkable presentations. This finding highlights that MA provides diagnostic resolution that cannot be consistently derived from clinical behavior alone, and supports its use as a standard component of the diagnostic workup in all pediatric CNS tumors referred with a histological diagnosis of ependymoma.

### Treatment results

**Fig. 2:**
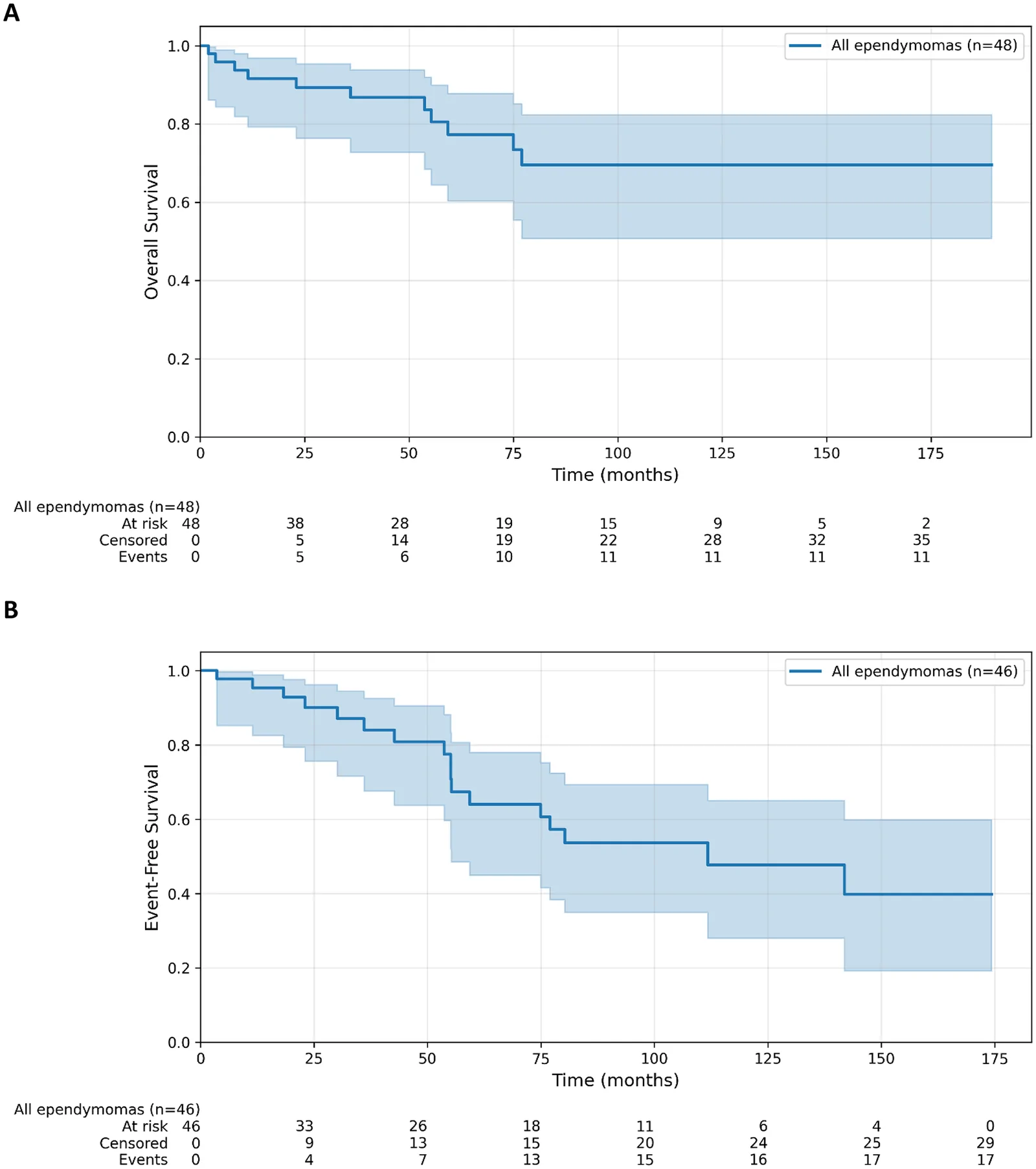
Overall (**A**) and Event-Free Survival (**B**) of all patients confirmed as ependymoma by methylation array. Two patients, who died as a consequence of primary surgery were excluded from the EFS analysis

Overall survival (OS) at 10 years for the entire ependymoma cohort was 69.6%, with event-free survival (EFS) of 47.7%. Two patients who died as a consequence of primary surgery were excluded from the EFS analysis (Fig. 2). Survival curves remained unchanged after excluding three patients with low methylation array calibration scores (Suppl. Figure 1).

When stratified by tumor location (excluding spinal tumors), posterior fossa ependymomas demonstrated 10-year OS of 56.6% and EFS of 39.8%, while supratentorial ependymomas showed 10-year OS of 83.1% and EFS of 59.1%. The differences between locations did not reach statistical significance for either OS (*p* = 0.197) or EFS (*p* = 0.415), although a trend toward better prognosis in supratentorial tumors was observed for OS (Fig. 3). Spinal ependymomas were not included in the location-based survival analysis because they represent a distinct group with an excellent prognosis. All 6 patients are alive: 5 are in complete remission and 1 experienced a relapse but achieved a second complete remission after surgery and radiotherapy. Their uniformly favorable course is already reflected in the 100% overall survival shown for this group in Suppl. Figure 2.

Extent of resection was the only factor associated with statistically significant survival differences. Among primary treatment responders (excluding spinal tumors and early postoperative deaths), patients achieving GTR (*n* = 35), including delayed GTR at second-look surgery) demonstrated dramatically superior 10-year OS compared to those with subtotal resection or biopsy only (*n* = 5), (75% vs. 40%, *p* = 0.017) (Fig. 3). Differences in survival among the ependymoma molecular subtypes were not statistically significant (Suppl. Figure 2).

Fig. 3OS (**A**) and EFS (**B**) in patients stratified according to the tumor location (excluding spinal) and OS (**C**) and EFS (**D**) by the extent of resection among primary treatment responders (excluding spinal tumors and early postoperative deaths)

**Figure:**
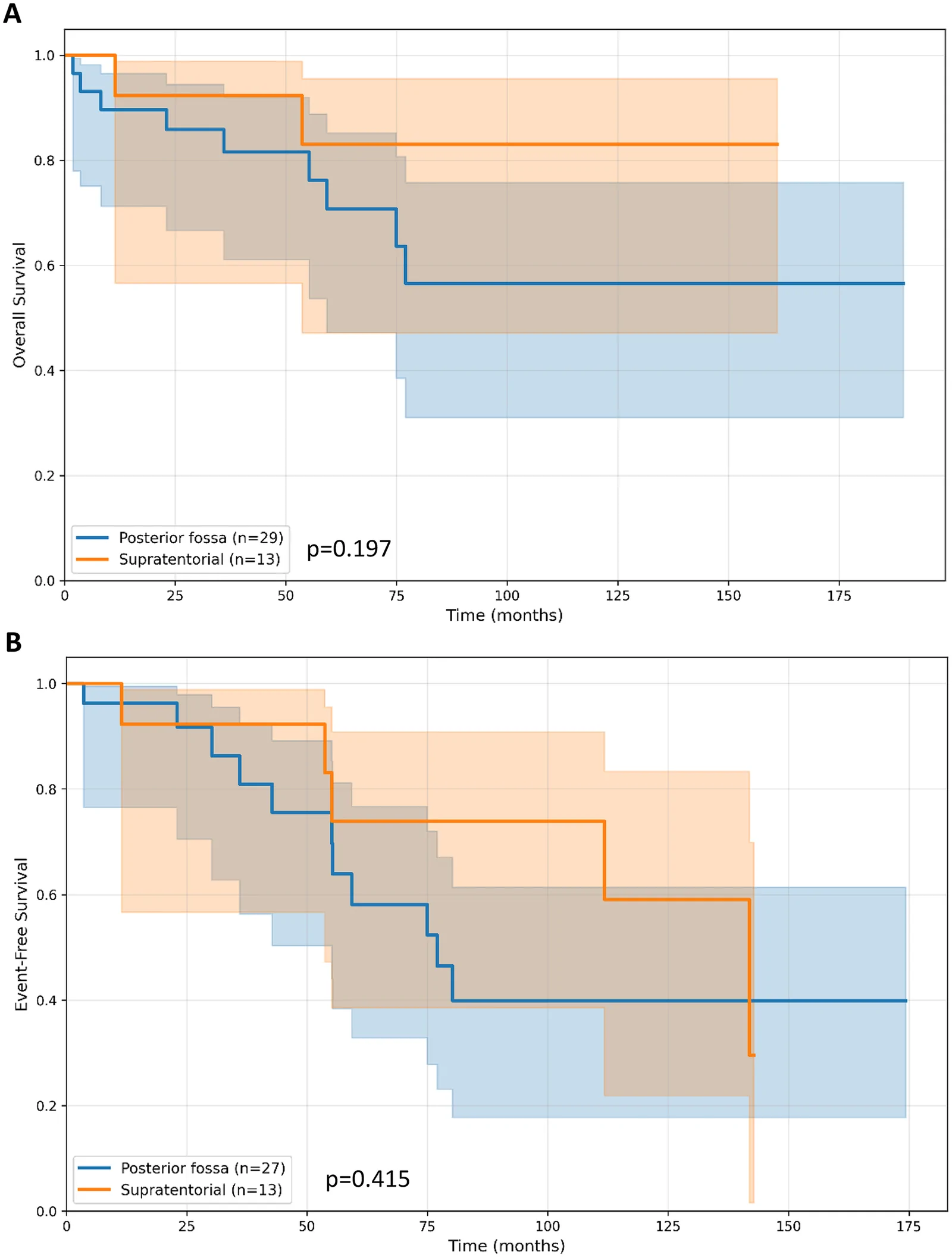

**Figure:**
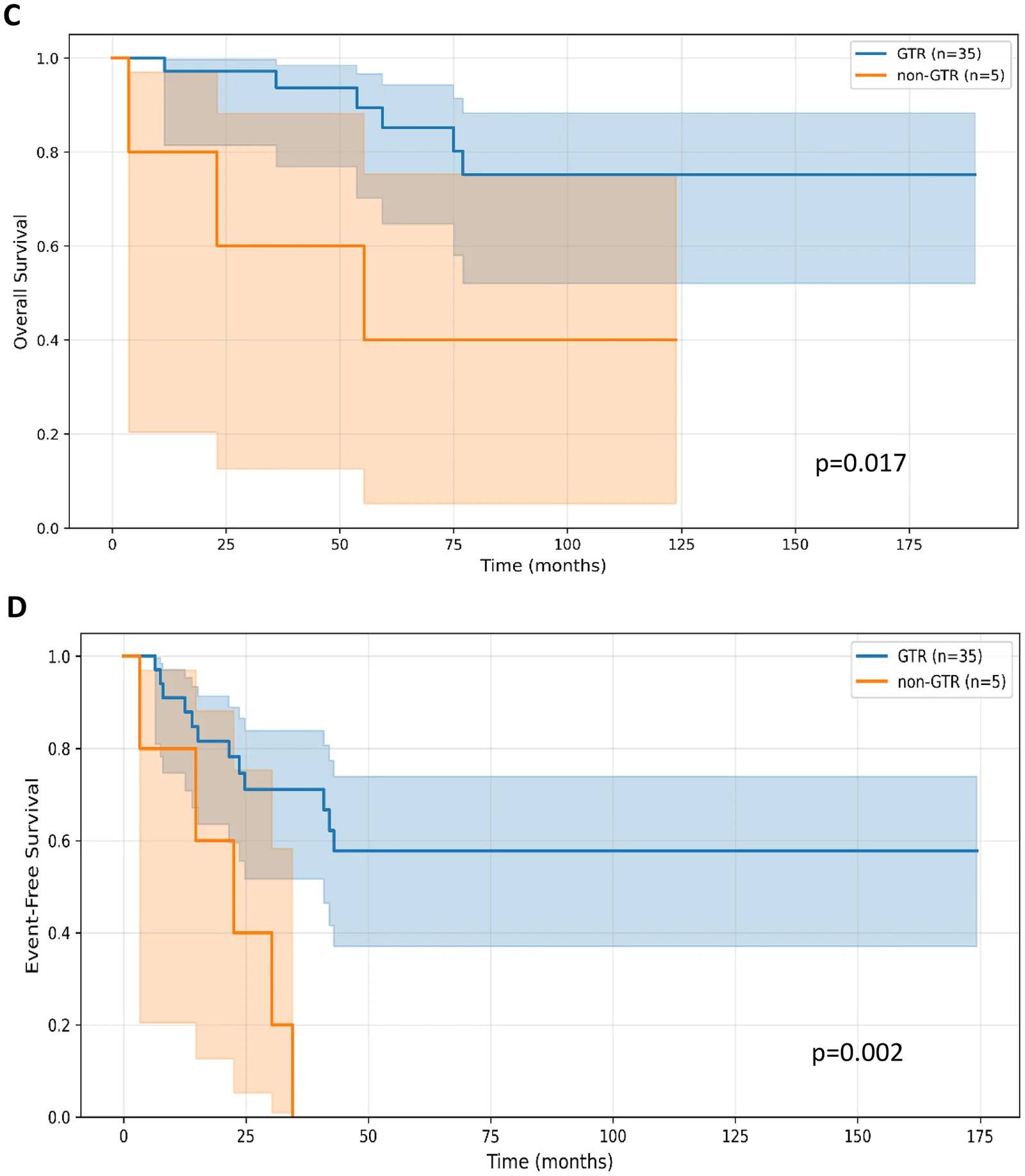


### Relapse characteristics and outcomes

Eighteen patients experienced disease relapse or progression, with a median time to relapse of 1.5 years (18.4 months) from diagnosis. The median age at diagnosis in this group was 2.2 years, substantially younger than patients without relapse (5.4 years), though this difference was not statistically significant (*p* = 0.65). The molecular distribution of relapsed patients included 10 PFA, 4 ST-ZFTA, 1 PFB, 2 ST-YAP1, and 1 MPE. Patterns of relapse were predominantly metastatic (*n* = 8) or local recurrence (*n* = 6), while four patients demonstrated progressive residual disease and never achieved GTR; two of these patients died shortly after initial surgery.

Management of relapsed disease was highly individualized but consistently prioritized achieving local control, primarily through re-resection to GTR when feasible, combined with radiotherapy and chemotherapy as clinically indicated (Suppl. Table 1). Two patients achieved long-term complete remission following GTR alone at relapse (ST-ZFTA) with ongoing remission extending 9 years. One patient with PFA died from radiotherapy-induced glioma after achieving prolonged disease control.

**Fig. 4:**
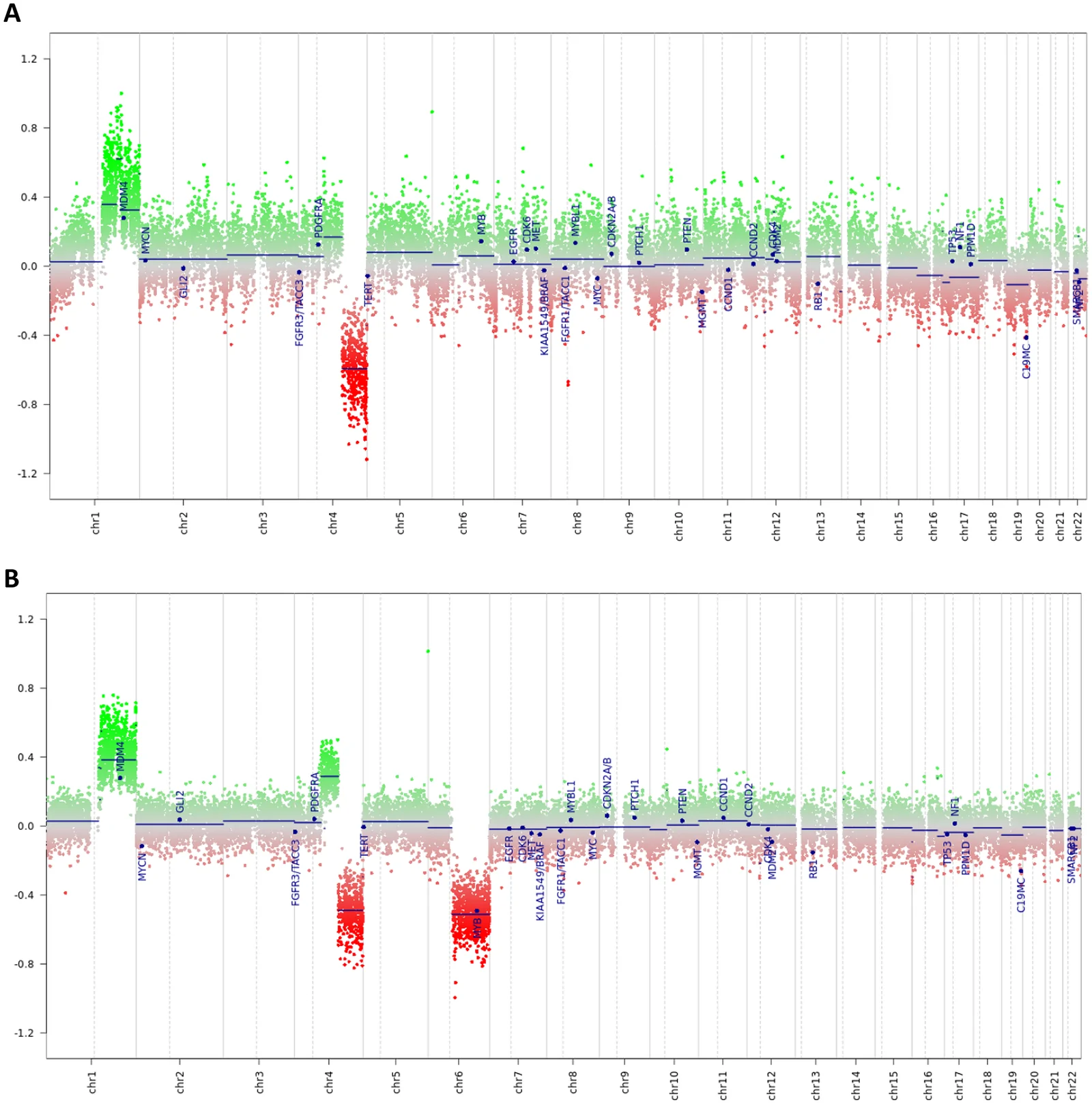
CNV plot of the samples of PFA patient (PRG12) from the time of diagnosis (**A**) with gain 1q and from the time of relapse (**B**) with acquired loss of 6q

Gain of 1q emerged as a particularly adverse prognostic marker: four of five PFA patients harboring this alteration experienced relapse despite standard therapy. However, outcomes after relapse were heterogeneous. While some patients exhibited aggressive disease courses with multiple recurrences despite intensive multimodal salvage therapy, others achieved meaningful disease control. Notably, one patient remains alive two years after relapse, and another (PRG 12)—who acquired an additional 6q loss at recurrence (Fig. 4)—is in complete remission three years after salvage treatment. These observations underscore the generally unfavorable but not uniformly fatal course of 1q gain-positive PFA ependymomas and highlight the biological complexity of recurrent disease.

## Discussion

The phenomenon whereby molecular profiling fundamentally revises a histopathological diagnosis is not unique to ependymoma. The most striking parallel comes from tumors formerly classified as CNS-PNET: retrospective DNA methylation profiling demonstrated that the majority of such cases represent biologically distinct entities — including high-grade gliomas, diffuse midline gliomas, ETMR, and ATRT — with dramatic prognostic consequences. In the CNS-PNET arm of COG ACNS0332, molecularly reclassified high-grade gliomas carried a 5-year OS of approximately 12%, compared with 78% in those whose embryonal tumor diagnosis was confirmed [11, 23]. A comparable evolution has occurred in medulloblastoma, where genome-wide methylation profiling defined four molecularly distinct subgroups with divergent prognoses and identified cases of misdiagnosis within large prospective cohorts [24, 25]. Our findings in ependymoma are consistent with this broader paradigm and underscore the necessity of methylation array analysis as an indispensable component of the diagnostic workup.

Our overall survival and event-free survival outcomes for confirmed ependymomas are broadly consistent with published series. The 10-year OS of 69.6% and 10-year EFS of 47.7% are comparable to results from large prospective cohorts, including the E-HIT2000 trial and ACNS0831 [26, 27]. Posterior fossa tumors showed worse outcomes than supratentorial tumors (10-year OS 56.6% vs. 83.1%, *p* = 0.197; 10-year EFS 47.1% vs. 49.9%, *p* = 0.949), without reaching statistical significance, likely reflecting limited sample size. Although size of the cohort did not allow for the multivariate analysis, extent of resection emerged as a significant prognostic factor — patients achieving GTR had a 10-year OS of 75% compared to 40% in those with residual disease (*p* = 0.017), consistent with the established central role of complete resection across all major ependymoma series. This underscores the importance of centralizing the care of such patients in pediatric neuro-oncology centers, where neurosurgeons have the necessary experience and the support of a multidisciplinary team. Outcomes stratified by molecular subgroup showed the expected trends — PFA tumors carried the worst prognosis (5/10-year EFS 44.7%), ST-ZFTA tumors intermediate outcomes (5-year EFS 58.3%), and spinal and myxopapillary ependymomas uniformly excellent outcomes — broadly mirroring E-HIT2000 data, although subgroup comparisons did not reach statistical significance given the small numbers involved [26, 27].

Meaningful comparison of outcomes in the misclassified cohort with published literature is not feasible, as these patients received treatment designed for ependymoma rather than for their true diagnosis. Notably, however, two patients reclassified as medulloblastoma Group 4 remain in sustained remission despite receiving substantially less intensive treatment than current standard of care for this subgroup — one treated with craniospinal irradiation (CSI) alone and one with CSI and oral etoposide only (Suppl. Table 2). While no conclusions can be drawn from two cases, this observation may warrant further attention in the context of biological heterogeneity within the medulloblastoma Group 4 category [28].

Routine implementation of DNA methylation profiling at diagnosis carries additional costs in terms of both resources and turnaround time. Our data, however, illustrate that these costs are justified: a substantial proportion of tumors diagnosed histologically as ependymoma represent biologically distinct entities requiring different treatment, and this misclassification carries direct clinical consequences. Early molecular assessment is therefore not an optional refinement but a diagnostic necessity [29, 30]. This position is increasingly reflected in prospective trial design. The E-HIT2000 cohort and Children´s Oncology Group in trial ACNS0831 have now established a molecularly informed risk stratification model that forms the framework for the next generation of European ependymoma trials [26, 27]. The ongoing SIOP Ependymoma II study incorporates centralized molecular classification as a prerequisite for treatment allocation, representing a paradigm shift from histology- to biology-driven therapy. We acknowledge that our cohort spans a 15-year period, during which supportive care, neurosurgical techniques, and radiological imaging have evolved, representing a limitations of our study. Our retrospective single-center cohort, despite its limitations, is consistent with this framework and supports the broader European effort to build molecularly annotated ependymoma registries as a basis for biologically stratified treatment strategies. 

## Supplementary Information

Below is the link to the electronic supplementary material.


Supplementary Material 1



Supplementary Material 2



Supplementary Material 3


## Data Availability

The datasets generated during and/or analyzed during the current study are available from the corresponding author on reasonable request.
