# Supplementary material for: Pediatric ependymoma in the molecular era: real-world experience with diagnostic correlation and long-term clinical outcomes
Source: J Neurooncol. 2026 Aug 5;179(1):30. doi: 10.1007/s11060-026-05740-y (PMC13442318; doi:10.1007/s11060-026-05740-y)
Supplement: Supplementary file 1 [file 11060_2026_5740_MOESM1_ESM.docx]

**Supplemental Figure 1 Overall (A) and Event-Free Survival (B) of all patients confirmed as ependymoma by methylation array excluding three patients with low calibration scores**

**A**

**B**

**Supplemental Figure 2 Overall Survival of ependymomas by methylation class (curves for ST-YAP1, MPE and Spinal overlap (all alive)**

p=0.062
