# Supplementary material for: Pediatric ependymoma in the molecular era: real-world experience with diagnostic correlation and long-term clinical outcomes
Source: J Neurooncol. 2026 Aug 5;179(1):30. doi: 10.1007/s11060-026-05740-y (PMC13442318; doi:10.1007/s11060-026-05740-y)
Supplement: Supplementary file 2 [file 11060_2026_5740_MOESM2_ESM.docx]

| Study ID | localisation | methylation class | relapse pattern | relapse surgery | relapse radiotherapy | relapse chemotherapy | status |
| --- | --- | --- | --- | --- | --- | --- | --- |
| **PRG1** | posterior fossa | Posterior Fossa Ependymoma Group A | metastatic | GTR | CSI | TMZ/Etoposide p.o. | DOD |
| **PRG7** | supratentorial | Supratentorial Ependymoma, ZFTA Fusion-Positive | local | GTR | no | no | CR |
| **PRG8** | supratentorial | Supratentorial Ependymoma, ZFTA Fusion-Positive | local | GTR | no | no | CR |
| **PRG11** | spine | Myxopapillary Ependymoma | metastatic | NTR | focal | no | CR |
| **PRG12** | posterior fossa | Posterior Fossa Ependymoma Group A | metastatic | GTR | CSI | Etoposide p.o. | CR |
| **PRG15** | posterior fossa | Posterior Fossa Ependymoma Group B | metastatic | GTR | RT | TMZ/Etoposide p.o. | DOD |
| **PRG16** | posterior fossa | Posterior Fossa Ependymoma Group A | local | GTR | no | UKCCSG CNS 9204 regime | DOD |
| **PRG24** | posterior fossa | Posterior Fossa Ependymoma Group A | local | GTR | focal | no | DOD |
| **PRG25** | posterior fossa | Posterior Fossa Ependymoma Group A | progressive residuum | no | no | no | DOD |
| **PRG28** | supratentorial | Supratentorial Ependymoma, YAP1 Fusion-Positive | local | GTR | no | TMZ/Etoposide p.o. | CR |
| **PRG30** | posterior fossa | Posterior Fossa Ependymoma Group A | metastatic | no | stereotactic | no | progressive disease |
| **PRG31** | supratentorial | Supratentorial ependymoma, ZFTA Fusion-Positive | metastatic | STR | no | VECC regime | DOD |
| **PRG32** | posterior fossa | Posterior Fossa Ependymoma Group A | progressive residuum | GTR - died of complications | no | no | DOD |
| **PRG34** | posterior fossa | Posterior Fossa Ependymoma Group A | metastatic | no | stereotactic | Etoposide p.o. | progressive disease |
| **PRG35** | supratentorial | Supratentorial Ependymoma, ZFTA Fusion-Positive | metastatic | GTR | focal | ICE regime | DOD |
| **PRG37** | supratentorial | Supratentorial Ependymoma, YAP1 Fusion-Positive | local | GTR | no | Vinblastin i.v. | CR |
| **PRG44** | posterior fossa | Posterior Fossa Ependymoma Group A | progressive residuum | NTR | stereotactic | no | DOD |
| **PRG47** | posterior fossa | Posterior Fossa Ependymoma Group A | progressive residuum | NTR | focal | no | progressive disease |
