# Supplementary material for: Pediatric ependymoma in the molecular era: real-world experience with diagnostic correlation and long-term clinical outcomes
Source: J Neurooncol. 2026 Aug 5;179(1):30. doi: 10.1007/s11060-026-05740-y (PMC13442318; doi:10.1007/s11060-026-05740-y)
Supplement: Supplementary file 3 [file 11060_2026_5740_MOESM3_ESM.docx]

| Study_ID | methylation class | methylation subgroup | metastatases | surgery | radiotherapy | chemotherapy | event | relapse tratment | status |
| --- | --- | --- | --- | --- | --- | --- | --- | --- | --- |
| **NE2** | Medulloblastoma Non-WNT/Non-SHH Activated | Medulloblastoma Group 4,Subclass VIII | yes | STR | CSI | Etoposide p.o. | no |  | CR |
| **NE5** | Medulloblastoma Non-WNT/Non-SHH Activated | Medulloblastoma Group 4,Subclass VII | no | GTR | focal | no | yes | RT CSI, TMZ/Etoposide p.o. | DOD |
| **NE6** | Medulloblastoma Non-WNT/Non-SHH Activated | Medulloblastoma Group 4,Subclass VIII | yes | GTR | CSI | no | no |  | CR |
| **NE9** | Medulloblastoma Non-WNT/Non-SHH Activated | Medulloblastoma Group 4,Subclass VII | no | GTR | focal | no | yes | ICE regime,RT CSI, TMZ/Etoposide p.o. | DOD |
